# A multicenter study of 1-year mortality and walking capacity after spinal fusion surgery for cervical fracture in elderly patients

**DOI:** 10.1186/s12891-022-05752-5

**Published:** 2022-08-20

**Authors:** Takeshi Sasagawa, Noriaki Yokogawa, Hiroyuki Hayashi, Hiroyuki Tsuchiya, Kei Ando, Hiroaki Nakashima, Naoki Segi, Kota Watanabe, Satoshi Nori, Kazuki Takeda, Takeo Furuya, Atsushi Yunde, Shota Ikegami, Masashi Uehara, Hidenori Suzuki, Yasuaki Imajo, Toru Funayama, Fumihiko Eto, Akihiro Yamaji, Ko Hashimoto, Yoshito Onoda, Kenichiro Kakutani, Yuji Kakiuchi, Nobuyuki Suzuki, Kenji Kato, Yoshinori Terashima, Ryosuke Hirota, Tomohiro Yamada, Tomohiko Hasegawa, Kenichi Kawaguchi, Yohei Haruta, Shoji Seki, Hitoshi Tonomura, Munehiro Sakata, Hiroshi Uei, Hirokatsu Sawada, Hiroyuki Tominaga, Hiroto Tokumoto, Takashi Kaito, Yoichi Iizuka, Eiji Takasawa, Yasushi Oshima, Hidetomi Terai, Koji Tamai, Bungo Otsuki, Masashi Miyazaki, Hideaki Nakajima, Kazuo Nakanishi, Kosuke Misaki, Gen Inoue, Katsuhito Kiyasu, Koji Akeda, Norihiko Takegami, Toshitaka Yoshii, Masayuki Ishihara, Seiji Okada, Yasuchika Aoki, Katsumi Harimaya, Hideki Murakami, Ken Ishii, Seiji Ohtori, Shiro Imagama, Satoshi Kato

**Affiliations:** 1 Department of Orthopaedic Surgery, Graduate School of Medical Sciences, Kanazawa University, 13-1 Takara-machi, Kanazawa, Ishikawa 920-8641 Japan; 2 Department of Orthopaedic Surgery, Toyama Prefectural Central Hospital, 2-2-78 Nishinagae, Toyama, Toyama 930-8550 Japan; 3 Department of Orthopaedic Surgery, Tonami General Hospital, Shintomicho 1-61, Tonami, Toyama 939-1395 Japan; 4 Department of Orthopedic Surgery, Graduate School of Medicine, Nagoya University, 65 Tsurumai-cho, Showa-ku, Nagoya, 466-8550 Japan; 5 Department of Orthopaedic Surgery, Keio University School of Medicine, 35 Shinanomachi, Shinjuku-ku, Tokyo, 160-8582 Japan; 6 Department of Orthopaedic Surgery, Japanese Red Cross Shizuoka Hospital, 8-2 Otemachi, Aoi-ku, Shizuoka, 420-0853 Japan; 7 Department of Orthopaedic Surgery, Graduate School of Medicine, Chiba University, 1-8-1 Inohana, Chuo-ku, Chiba, Chiba 260-8670 Japan; 8 Department of Orthopaedic Surgery, Shinshu University School of Medicine, 3-1-1 Asahi, Matsumoto, Nagano 390-8621 Japan; 9 Department of Orthopaedic Surgery, Yamaguchi University Graduate School of Medicine, 1-1-1 Minami-kogushi, Ube city, Yamaguchi 755-8505 Japan; 10 Department of Orthopaedic Surgery, Faculty of Medicine, University of Tsukuba, 1-1-1 Tennodai, Tsukuba, Ibaraki 305-8575 Japan; 11 Department of Orthopaedic Surgery, Graduate School of Comprehensive Human Sciences, University of Tsukuba, 1-1-1 Tennodai, Tsukuba, Ibaraki 305-8575 Japan; 12 Department of Orthopaedic Surgery, Ibaraki Seinan Medical Center Hospital, 2190, Sakaimachi, Sashima, Ibaraki 306-0433 Japan; 13 Department of Orthopaedic Surgery, Tohoku University Graduate School of Medicine, 1-1 Seiryo-machi, Aoba-ku, Sendai, Miyagi 980-8574 Japan; 14 Department of Orthopaedic Surgery, Kobe University Graduate School of Medicine, 7-5-1 Kusunoki-cho, Chuo-ku, Kobe, 650-0017 Japan; 15 Department of Orthopaedic Surgery, Nagoya City University Graduate School of Medical Sciences, 1 Kawasumi, Mizuho-cho, Mizuho-ku, Nagoya, 467-8601 Japan; 16 Department of Orthopaedic Surgery, Sapporo Medical University, South 1-West 16-291, Chuo-ku, Sapporo, 060-8543 Japan; 17 Department of Orthopaedic Surgery, Matsuda Orthopedic Memorial Hospital, North 18-East 4-1 Kita-ku, Sapporo, 001-0018 Japan; 18 Department of Orthopaedic Surgery, Hamamatsu University School of Medicine, 1-20-1, Handayama, Higashi-ku, Hamamatsu City, Shizuoka, 431-3192 Japan; 19 Department of Orthopaedic Surgery, Nagoya Kyoritsu Hospital, 1-172 Hokke, Nakagawa-ku, Nagoya-shi, Aichi, 454-0933 Japan; 20 Department of Orthopaedic Surgery, Graduate School of Medical Sciences, Kyushu University, 3-1-1 Maidashi Higashi-ku, Fukuoka, 812-8582 Japan; 21 Department of Orthopaedic Surgery, Faculty of Medicine, University of Toyama, 2630 Sugitani, Toyama, Toyama 930-0194 Japan; 22 Department of Orthopaedics, Graduate School of Medical Science, Kyoto Prefectural University of Medicine, Kawaramachi-Hirokoji, Kamigyo-ku, Kyoto, 602-8566 Japan; 23 Department of Orthopaedics, Saiseikai Shiga Hospital, 2-4-1 Ohashi Ritto, Shiga, 520-3046 Japan; 24 Department of Orthopaedic Surgery, Nihon University Hospital, 1-6 Kanda-Surugadai, Chiyoda-ku, Tokyo, 101-8393 Japan; 25 Department of Orthopaedic Surgery, Nihon University School of Medicine, 30-1 Oyaguchi Kami-cho, Itabashi-ku, Tokyo, 173-8610 Japan; 26 Department of Orthopaedic Surgery, Graduate School of Medical and Dental Sciences, Kagoshima University, 8-35-1 Sakuragaoka, Kagoshima, 890-8520 Japan; 27 Department of Orthopaedic Surgery, Osaka University Graduate School of Medicine, 2-2 Yamadaoka, Suita, Osaka 565-0871 Japan; 28 Department of Orthopaedic Surgery, Graduate School of Medicine, Gunma University, 3-39-22 Showa, Maebashi, Gunma 371-8511 Japan; 29 Department of Orthopaedic Surgery, The University of Tokyo Hospital, 7-3-1 Hongo, Bunkyo-ku, Tokyo, 113-8655 Japan; 30 Department of Orthopaedic Surgery, Osaka City University Graduate School of Medicine, 1-4-3 Asahimachi, Abeno-ku, Osaka-city, Osaka, 545-8585 Japan; 31 Department of Orthopaedic Surgery, Graduate School of Medicine, Kyoto University, 54 Shogoin-Kawaracho, Sakyo-ku, Kyoto, Kyoto Japan; 32 Department of Orthopaedic Surgery, Faculty of Medicine, Oita University, 1-1 Idaigaoka, Hasama-machi, Yufu-shi, Oita, 879-5593 Japan; 33 Department of Orthopaedics and Rehabilitation Medicine, Faculty of Medical Sciences, University of Fukui, 23-3 Matsuoka Shimoaizuki, Eiheiji-cho, Yoshida-gun, Fukui, 910-1193 Japan; 34 Department of Orthopedics, Traumatology and Spine Surgery, Kawasaki Medical School, Matsushima, Kurashiki, Okayama 577701-0192 Japan; 35 Department of Orthopaedic Surgery, Kitasato University School of Medicine, 1-15-1, Kitazato, Minami-ku, Sagamihara, Kanagawa 252-0374 Japan; 36 Department of Orthopaedic Surgery, Kochi Medical School, Kochi University, KohasuNankoku, Oko-cho 783-8505 Japan; 37 Department of Orthopaedic Surgery, Mie University Graduate School of Medicine, 2-174 Edobashi, Tsu city, Mie, 514-8507 Japan; 38 Department of Orthopaedic Surgery, Tokyo Medical and Dental University, Yushima 1-5-45, Bunkyo-Ku, Tokyo, 113-8519 Japan; 39 Department of Orthopaedic Surgery, Kansai Medical University Hospital, 2-3-1 Shinmachi, Hirakata, Osaka 573-1191 Japan; 40 Department of Orthopaedic Surgery, Eastern Chiba Medical Center, 3-6-2 Okayamadai, Togane, Chiba 283-8687 Japan; 41 Department of Orthopaedic Surgery, Kyushu University Beppu Hospital, Tsurumibaru, Beppu, Oita, 874-0838 Japan; 42 Department of Orthopaedic Surgery, School of Medicine, International University of Health and Welfare, 852 Hatakeda, Narita, Chiba 286-0124 Japan; 43 Department of Orthopaedic Surgery, International University of Health and Welfare Narita Hospital, 852 Hatakeda, Narita, Chiba 286-0124 Japan; 44 Department of Orthopaedic Surgery and Spine and Spinal Cord Center, International University of Health and Welfare Mita Hospital, 1-4-3 Mita, Minato-ku, Tokyo, 108-8329 Japan

**Keywords:** Cervical fracture, Spinal fusion surgery, Elderly, Mortality, Walking capacity

## Abstract

**Background:**

The 1-year mortality and functional prognoses of patients who received surgery for cervical trauma in the elderly remains unclear. The aim of this study is to investigate the rates of, and factors associated with mortality and the deterioration in walking capacity occurring 1 year after spinal fusion surgery for cervical fractures in patients 65 years of age or older.

**Methods:**

Three hundred thirteen patients aged 65 years or more with a traumatic cervical fracture who received spinal fusion surgery were enrolled. The patients were divided into a survival group and a mortality group, or a maintained walking capacity group and a deteriorated walking capacity group. We compared patients’ backgrounds, trauma, and surgical parameters between the two groups. To identify factors associated with mortality or a deteriorated walking capacity 1 year postoperatively, a multivariate logistic regression analysis was conducted.

**Results:**

One year postoperatively, the rate of mortality was 8%. A higher Charlson comorbidity index (CCI) score, a more severe the American Spinal Cord Injury Association impairment scale (AIS), and longer surgical time were identified as independent factors associated with an increase in 1-year mortality. The rate of deterioration in walking capacity between pre-trauma and 1 year postoperatively was 33%. A more severe AIS, lower albumin (Alb) and hemoglobin (Hb) values, and a larger number of fused segments were identified as independent factors associated with the increased risk of deteriorated walking capacity 1 year postoperatively.

**Conclusions:**

The 1-year rate of mortality after spinal fusion surgery for cervical fracture in patients 65 years of age or older was 8%, and its associated factors were a higher CCI score, a more severe AIS, and a longer surgical time. The rate of deterioration in walking capacity was 33%, and its associated factors were a more severe AIS, lower Alb, lower Hb values, and a larger number of fused segments.

## Background

In step with the overall aging of the population, the frequency of occurrence of cervical spinal fractures in the elderly has increased in recent years [1, 2]. Cervical spine fractures may occur in isolation or in conjunction with a spinal cord injury and are relatively common among older adults, whose susceptibility may increase with minor trauma. Cervical spine fractures represent an important cause of mortality among adults aged 65 years or older [1, 2]. The mortality associated with cervical spinal fractures in elderly patients exceeds that in younger patients [3]. Compared with a hip fracture, a common type of fracture in the elderly, patients with cervical fractures had a greater mortality than those with hip fractures [1]. Surgical treatment for cervical trauma is a more invasive option compared with conservative treatment. Therefore, it is important to understand mortality and functional outcomes after surgical treatment for cervical trauma.

In previous reports that have explored the relationship between mortality and cervical spinal fractures in the elderly, only in-hospital mortality has been discussed [2–5], and only a few reports have referred to 1 year mortality [6, 7]. Furthermore, most of the previous reports included both surgical and conservative treatments [2–6], and few reports specify the mortality of patients treated with surgery alone [7]. The 1-year mortality of patients who received surgery for cervical trauma remains unclear. Moreover, to our knowledge, although functional outcomes after surgery for cervical trauma are important, 1-year postoperative functional prognoses remain unreported. The aim of this study is to investigate the rates of, and factors associated with mortality and any changes in walking capacity occurring 1 year after spinal fusion surgery for cervical fractures in patients 65 years of age or older.

## Materials and methods

### Study design and ethical considerations

This study retrospectively analyzed multicenter registry data collected by the Japan Association of Spine Surgeons with Ambition (JASA). The institutional review board of the representative facility reviewed and approved this study. No funds were received in support of this study. No benefits in any form have been or will be received from a commercial party related directly or indirectly to the subject of this manuscript.

### Patient population

Patients aged 65 years or more with a traumatic cervical fracture who received spinal fusion surgery from February 2010 to August 2019 at 68 institutions registered with JASA were considered for inclusion in this study. Patients who received surgery more than 90 days postinjury, or those for whom missing data pertaining to the type of surgery and level(s) of fusion were missing, were excluded. Of the 418 patients who were eligible for study participation, 105 of them were lost to follow-up 1 year postoperatively (follow-up rate was 75%) and 313 patients were enrolled.

### Data collection

#### Patients’ background data

Data for each patient, including age at time of injury, gender, height, weight, pre-trauma walking capacity, Charlson Comorbidity Index (CCI) score [8], and blood test results at the first preoperative visit were collected. We defined the main walking style in everyday life as walking capacity.　Patients’ walking capacities were divided into four grades: independent, able to walk with a T-cane, able to walk with a walker, or inability to walk. Blood tests were used to measure albumin (Alb) and hemoglobin (Hb).

#### Trauma data

Collected radiographic data included the fracture level(s), presence of facet interlocking, and comorbid major organ injury. Comorbid major organ injury was defined as other trauma requiring surgery, hemothorax requiring a chest drain, or brain injury with consciousness disturbance. Neurological impairment was accessed using the American Spinal Cord Injury Association (ASIA) impairment scale (AIS), ranging from Grade A (complete impairment) to Grade E (normal function) [9]. In patients with AIS A to D, spinal cord injury type (transverse or central) was also investigated.

#### Surgical data

The surgical data documented included the surgical approach (anterior, posterior, or combined), number of fused levels, presence of occipitocervical fusion, surgical bleeding (mL), and surgical time (min).

### Operative outcomes

Collected operative outcomes included intraoperative and postoperative complications, patient mortality, and walking capacity 1 year postoperatively.

### Statistical analysis

All 313 patients were divided into a survival group and a mortality group. We also divided the patients who were able to walk before injury and survived 1 year postoperatively into a ‘maintained walking capacity’ group and a ‘deteriorated walking capacity’ group. Deteriorated walking capacity applied when a patient’s walking capacity decreased by at least one grade between pre-trauma and 1 year postoperatively. We compared patients’ backgrounds, trauma, and surgical parameters between the two groups. All data are expressed as mean ± standard deviation. A Mann–Whitney U test, chi square test, or Fisher’s exact test was used to compare each item. To identify factors associated with mortality or a deteriorated walking capacity 1 year postoperatively, a multivariate logistic regression analysis was conducted in which mortality or a deteriorated walking capacity were used as a dependent variable. Items that were significantly different by univariate analysis were independent variables. Differences were considered significant at *P* < 0.05. All statistical analyses were performed using IBM SPSS Statistics for Windows (version 22; IBM Corp, Armonk, NY).

## Results

The demographic data for 313 cases are presented in Table 1. There were 201 men and 112 women of mean age 74.6 years in this study. Of the 313 patients we assessed, 99% were able to walk before injury. The most common site for cervical fractures was C6‒7 (46%), followed by C3‒5 (43%), and C1‒2 (31%). The frequency of facet interlocking was 28%, and a spinal cord injury occurred in 51% of patients. The type of spinal cord injury in 158 patients with AIS A-D was transverse in 88 patients (56%) and central in 70 patients (44%). Surgery was most frequently via the posterior approach (87%). The intraoperative and postoperative complications of the 313 cases are presented in Table 2. The most frequent intraoperative complication was an iatrogenic dural tear (2%), and the most frequent postoperative complication was pneumonia (11%).

**Table 1:** Patient characteristics and demographics

Parameter^a^	n	N (%)^b^
Patients, n		313
Age, years		74.6 ± 6.2
Gender (male: female), n		201:112
Height, cm	298	158.9 ± 9.7
Weight, kg	300	55.8 ± 10.3
Pre-trauma walking capacity, n		
Independent / T-cane / walker / inability to walk		291 / 13 / 5 / 4
Charlson comorbidity index	307	0.6 ± 1.0
Blood test data, g/dL		
Albumin	267	3.7 ± 0.5
Hemoglobin	309	12.6 ± 1.9
Level of fracture, n (%)		
C1‒2 / C3‒5 / C6‒7		98 (31) / 113 (36) / 144 (46)
Facet interlocking, n (%)		89 (28)
ASIA Impairment Scale, n (%)		
A / B / C / D / E	312	27 (9) / 14 (5) / 43 (14) / 74 (24) / 154 (49)
Comorbid major organ injury, n (%)		31 (10)
Surgery		
Anterior / Posterior / Combined, n (%)		30 (10) /273 (87) / 10 (3)
Number of fused segments		2.4 ± 1.8
Occipitocervical fusion, n (%)		16 (5)
Surgical bleeding, mL	291	174 ± 71
Surgical time, min	288	247 ± 387

*ASIA* American Spinal Injury Association

^a^ Data are the mean ± standard deviation unless otherwise shown

^b^ Results for a denominator of *N* = 313, unless otherwise indicated by n in middle column

**Table 2:** Overall operative outcomes

Outcomes	*N* = 313
Intraoperative complications, n (%)	
Dural tear	7 (2)
Vertebral artery injury	4 (1)
Spinal cord injury	1 (< 1)
Postoperative complications, n (%)	
Pneumonia	33 (11)
Delirium	30 (10)
Urinary tract infection	27 (9)
Surgical site infection	6 (2)
Cerebral infarction	5 (2)
Instrumentation failure	4 (1)
Cerebrospinal fluid leakage	4 (1)
Pulmonary embolism	3 (1)
Epidural hematoma	1 (< 1)
1-year mortality, %	
Overall	8
ASIA Impairment Scale A / B / C / D / E	44 / 14 / 5 / 1 / 5
Walking capacity deterioration 1-year postoperatively^a,^ %	
Overall	33
ASIA Impairment Scale A / B / C / D / E	100 / 100 / 63 / 21 / 18

*ASIA* American Spinal Injury Association

^a^ *n* = 284 patients who can walk before injury

### Life prognosis

One year postoperatively, 25 out of 313 patients had died and the rate of mortality was 8% (Table 2). Death occurred within one month in 12 of 25 patients (48%) and within six months in 20 of 25 patients (80%). The causes of death were respiratory complications (respiratory failure, asphyxia, pneumonia) in 12 cases, cerebral infarction in 3 cases, gastrointestinal complications in 2 cases, malignancy in 2 cases, heart failure in 2 cases, pulmonary embolism in 1 case, massive intraoperative bleeding in 1 case, and details unknown in 2 cases. A comparison between the survival group (*n* = 288) and the mortality group (*n* = 25) revealed that being male, being assessed as having a higher CCI score, a severe AIS, and/or a longer surgical time were more significantly associated with the mortality group than the survival group (Table 3). A multivariate logistic regression analysis was conducted to identify factors associated with 1-year mortality. A higher CCI score (odds ratio [OR] = 2.046, 95% confidence interval [CI]: 1.398–2.993), more severe AIS (OR = 2.205, 95% CI: 1.586–3.065), and longer surgical time (OR = 1.009, 95% CI: 1.002–1.015) were identified as independent factors associated with an increase in 1-year mortality (Table 4).

**Table 3:** Comparison of admission data between survival and mortality groups 1 year postoperatively

	n	Survival group (*n* = 288)	n	Mortality group (n = 25)	*P*
Patient background					
Age, years		74.5 ± 6.3		76.6 ± 5.4	0.07
Gender (male / female), n		179 / 109		22 / 3	0.01*
Height, cm	274	158.7 ± 9.7	24	161.5 ± 9.9	0.19
Weight, kg	277	55.7 ± 10.3	24	56.8 ± 10.8	0.78
Pre-trauma walking capacity, n					
Independent / T-cane / walker / inability to walk		269 / 12 / 3 / 4		22 / 1 / 2 / 0	0.14
Charlson comorbidity index	282	0.5 ± 1.0		1.3 ± 1.6	< 0.01*
Blood test data, g /dL					
Albumin	247	3.7 ± 0.5	20	3.6 ± 0.4	0.16
Hemoglobin	285	12.7 ± 1.9	24	12.1 ± 2.3	0.33
Level of fracture, n (%)					
C1‒2		93 (32)		5 (20)	0.20
C3‒5		102 (35)		11 (44)	0.39
C6‒7		130 (45)		14 (56)	0.30
Facet interlocking, n (%)		81 (28)		8 (32)	0.68
ASIA Impairment Scale, n (%)					< 0.01*
A / B / C / D / E	287	15 (5) /12 (4) / 41 (14) / 73 (25) / 146 (51)		12 (48) / 2 (8) / 2 (8) / 1 (4) / 8 (32)	
Comorbid major organ injury, n (%)		30 (10)		1 (4)	0.26
Surgery					
Anterior / Posterior / Combined, n (%)		28 (10) / 251 (87) / 9 (3)		2 (8) / 22 (88) / 1 (4)	0.94
Number of fused segments		2.3 ± 1.7		3.0 ± 2.0	0.10
Occipitocervical fusion, n (%)		15 (5)		1 (4)	0.63
Surgical bleeding, mL	266	228 ± 304	22	481 ± 886	0.10
Surgical time, min	268	171 ± 68	23	217 ± 83	< 0.01*

*ASIA* American Spinal Injury Association

^a^ Data are the mean ± standard deviation unless otherwise shown

^b^ Results for denominator *n* = 288 (survivor group) or *n* = 25 (mortality group), unless otherwise indicated in the column to the left of each of these group results, respectively

^*^P < 0.05

**Table 4:** Multivariate logistic regression analysis of associated factors of mortality 1 year postoperatively

Variables	OR	95% CI	*P*
Gender: male	0.419	0.109–1.611	0.21
Charlson comorbidity index	2.046	1.398–2.993	< 0.01*
ASIA Impairment Scale	2.205	1.586–3.065	< 0.01*
Surgical time (min)	1.009	1.002–1.015	0.01*

*OR* Odds ratio, *CI* Confidence interval, *ASIA* American Spinal Injury Association

The ASIA Impairment Scale is treated as an ordinal variable (A-E)

^*^P < 0.05

### Walking capacity

Of 313 patients, 284 patients were able to walk before injury and survived 1 year postoperatively. Among these 284 patients, 93 patients (33%) exhibited a deterioration in their pre-trauma walking capacity 1 year postoperatively (Table 2). The rate of walking capacity deterioration 1-year postoperatively by AIS was 100% for AIS A/B (all cases with transverse spinal cord injuries), 63% (transverse type 72%, central type 47%) for AIS C, 21% (transverse type 44%, central type 13%) for AIS D, and 18% for AIS E. A comparison between the maintained walking capacity group (*n* = 191) and the deteriorated walking capacity group (*n* = 93) revealed the values of Alb and Hb, and the frequency of C1‒2 fractures were significantly less in the deteriorated walking capacity group. In contrast, the frequency of C3‒5 fractures, severe AIS, number of fused segments, and surgical bleeding were significantly higher in the deteriorated walking capacity group than the maintained walking capacity group (Table 5). A multivariate logistic regression analysis was conducted to identify factors associated with deteriorated walking capacity 1 year postoperatively. A more severe AIS (OR = 4.092, 95% CI: 2.767–6.052), lower Alb (OR = 0.341, 95% CI: 0.172–0.672), lower Hb (OR = 0.797, 95% CI: 0.660–0.961) values and a larger number of fused segments (OR = 1.241, 95% CI: 1.023–1.506) were identified as independent factors associated with the increased risk of deteriorated walking capacity 1 year postoperatively (Table 6).

**Table 5:** Comparison of admission data between maintained and deteriorated walking capacity groups 1 year postoperatively

	n	Maintained walking capacity group (*n* = 191)	n	Deteriorated walking capacity group (*n* = 93)	*P*
Patient background					
Age, years		74.3 ± 6.2		74.6 ± 6.4	0.78
Gender (male / female), n		111 / 80		64 / 29	0.08
Height, cm	185	158.4 ± 9.7	85	159.3 ± 9.6	0.36
Weight, kg	186	55.0 ± 10.0	87	57.5 ± 10.9	0.07
Pre-trauma walking capacity, n					
Independent / T-cane / walker		183 / 6 / 2		86 / 6 / 1	0.35
Charlson comorbidity index	189	0.5 ± 1.0	89	0.6 ± 0.9	0.20
Blood test data, g /dL					
Albumin	156	3.8 ± 0.5	87	3.6 ± 0.6	< 0.01*
Hemoglobin	188	12.9 ± 1.9	84	12.3 ± 1.8	0.02*
Level of fracture, n (%)					
C1‒2		71 (37)		22 (24)	0.02*
C3‒5		59 (31)		41 (44)	0.03*
C6‒7		86 (45)		42 (45)	0.98
Facet interlocking, n (%)		53 (28)		28 (30)	0.68
ASIA Impairment Scale, n (%)					< 0.01*
A / B / C / D / E		0 / 0 / 15 (8) / 57 (30)/ 119 (62)		15 (16) / 11 (12) / 25 (27) / 15 (16) / 27 (29)	
Comorbid major organ injury, n (%)		16 (8)		14 (15)	0.09
Surgery					
Anterior / Posterior / Combined, n (%)		20 (10) / 164 (86) / 7 (4)		7 (8) / 84 (90) / 2 (2)	0.56
Number of fused segments		2.1 ± 1.6		2.6 ± 1.9	0.01*
Occipitocervical fusion, n (%)		8 (4)		7 (8)	0.18
Surgical bleeding, mL	176	187 ± 220	86	291 ± 363	0.01*
Surgical time, min	181	168 ± 72	83	176 ± 60	0.25
Walking capacity 1 year postoperatively, n					
Independent / T-cane / walker / inability to walk		184 / 6 / 1 / 0		0 / 32 / 33 / 28	

^a^ Data are the mean ± standard deviation unless otherwise shown

^b^ Results for denominator of *n* = 191 (maintained walking capacity group) or *n* = 93 (deteriorated walking capacity group), unless otherwise indicated by in the column to the left of each of these group results, respectively

*ASIA* American Spinal Injury Association

^*^P < 0.05

**Table 6:** Multivariate logistic regression analysis of associated factors of deteriorated walking capacity 1 year postoperatively

Variables	OR	95% CI	*P*
Albumin (g/dL)	0.341	0.172 – 0.672	< 0.01*
Hemoglobin (g/dL)	0.797	0.660 – 0.961	0.02*
Fracture at C1‒2	0.489	0.210 – 1.140	0.10
Fracture at C3‒5	0.263	0.323 – 1.362	0.26
ASIA Impairment Scale	4.092	2.767 – 6.052	< 0.01*
Fused segments	1.241	1.023 – 1.506	0.03
Surgical bleeding (mL)	1.001	1.000 – 1.002	0.19

*OR* Odds ratio, *CI* Confidence interval, *ASIA* American Spinal Injury Association

The ASIA Impairment Scale is treated as an ordinal variable (A-E)

^*^P < 0.05

## Discussion

Based on the present study, we report a 1-year mortality rate of 8% after spinal fusion surgery for cervical fractures in patients 65 years of age or older. In previous reports of cervical spinal fractures in the elderly, the rates of mortality coexistent with a spinal cord injury were 7‒53% [2–6]. In our study, 51% of cervical fractures were associated with a spinal cord injury. Thus, our results reflect cervical fractures with a high rate of concomitant spinal cord injury. This can be attributed to the fact that the present study comprised patients who received spinal fusion surgery. Previous reports of mortality associated with cervical spine fractures in the elderly have referred to an in-hospital mortality rate of 8‒14% [2–5], and a 1-year mortality rate of 28‒29% [6, 7]. Most of those previous reports included both surgical and conservative treatments [2–6]. Although Sander et al. have reported on postsurgical mortality, they noted decompression surgery was included in addition to fusion surgery [7]. In contrast, our study comprised only patients who received spinal fusion surgery for cervical fractures. The best studied relationship between injury type and mortality rate is for hip fractures. The reported 1-year mortality rate after hip fracture in the elderly is 10‒30% [10, 11]. Patients with a cervical fracture incur a greater risk of mortality compared with those who sustain a hip fracture [1]. However, we did not demonstrate this phenomenon in the current study.

Our study did show that a higher CCI score, more severe AIS, and a longer surgical time were identified as independent factors associated with increasing 1-year mortality. Previously, whether a greater number of comorbidities was associated with mortality or not is controversial in the elderly with cervical fractures [6, 12]. The results of this study indicated that a greater number of comorbidities was associated with an increased mortality risk in patients treated surgically. Previously, a neurological deficit has been linked to mortality after a cervical fracture in the elderly [1–3, 6, 13] and mortality has been correlated with the severity of a neurological deficit [13, 14]. The results of our study supported the same finding. Daneshvar et al. demonstrated that an injury at or above C4 had a 7.1 times higher risk of mortality compared with injuries below C4 when spinal cord injuries were related to cervical spine fractures [13]. The logical connection to consider is that the more severe the spinal cord injury, the greater the impact on respiratory muscle function and hence an increased risk for mortality.

We included surgical factors in our investigation because our study comprised patients who were treated surgically. As a result, a longer surgical time was identified as an independent factor associated with increasing 1-year mortality. There are two possible explanations. First, a cervical fracture requiring a long surgical time is typically indicative of severe trauma leading to poor general condition. Second, based on our result that 48% of those in the mortality group died within one month postoperatively, surgical invasiveness is considered to be related to mortality in the immediate postoperative window. Some reports indicate that surgical invasiveness for spinal trauma in the acute phase is related to the mortality [6, 15]. Also, greater surgical invasiveness during spinal surgery increases postoperative complications, particularly in the elderly [16]. In our study results pneumonia was the most frequent postoperative complication reported. In addition, respiratory complications were the most common cause of death. Bokhari et al. reported that the occurrence of pneumonia is more frequent during surgical treatment than conservative treatment in elderly patients with cervical fractures [17]. In short, there is a possibility that surgical invasiveness gave rise to a decline in the general condition of the patients, resulting in poor respiratory function leading to mortality. In terms of surgical factors, in our study we were also able to demonstrate that surgical time was a more important factor than surgical approach, surgical bleeding, or number of fused levels.

The rate of deterioration in walking capacity between the time of injury and 1 year postoperatively was 33% in this study. To our knowledge, there are no reports of functional outcomes 1 year after spinal fusion surgery for elderly patients with cervical fractures. With regard to hip fractures in the elderly, the reported 1-year postoperative rate of deterioration in walking capacity is 26‒61% [18–20]. Therefore, the rate of deterioration in walking capacity in the elderly with a cervical fracture in our study was comparable to that reported for hip fracture.

A more severe AIS, lower Alb, lower Hb values and a larger number of fused segments were identified as independent factors associated with an increased risk of deteriorated walking capacity 1 year postoperatively. Previously, neurological deficits have been related to poor functional outcomes [5]. Our study also demonstrated that the severity of neurological deficits was related to poor functional outcomes for as long as 1 year postoperatively. Reports have indicated that recovery from a spinal cord injury is poor in the elderly population [13]. Moreover, even when neurological function recovered, elderly patients experience difficulties translating improvements in a neurological outcome into functional changes [21]. For these reasons, the severity of a neurological deficit at the time of injury is considered to be strongly associated with a deterioration in walking capacity 1 year postoperatively.

Serum Alb has traditionally been used as a marker for poor health and nutrition. In the elderly with hip fractures, hypoalbuminemia or poor nutrition is related to poor functional outcomes [22, 23]. Hypoalbuminemia also has been reported to be associated with poor functional outcomes in cervical spinal cord injury [24]. In short hypoalbuminemia is considered to lead to poor recovery of neuromuscular function. We demonstrated that low preoperative Alb values were associated with a poor functional outcome in the elderly with a cervical fracture, as well.

A low level of preoperative Hb has been associated with a poor functional outcome according to some reports of hip fractures in the elderly [25, 26]. Low preoperative Hb values were associated with poor functional outcomes in the elderly with cervical fracture in our study also. A possible explanation for the relationship between low Hb values and poor functional outcomes is that Hb status could be a marker of an underlying comorbidity [25]. Anemia has been observed to be a risk factor for the frailty phenotype in the elderly [27]. Thus, low Hb values preoperatively could be a reflection of greater frailty associated with a poor functional outcome [25].

A larger number of fused segments was identified as an independent factor associated with the increased risk of a deteriorated walking capacity one year postoperatively. Previously, it was reported that a larger number of fused segments was associated with disability and compromises to activities of daily living [28]. Furthermore, whole cervical spine fixation increases stride time and decreases stride length. Fixation reduces motions between the shoulder girdle and the trunk, and between the trunk and the pelvis, and decreases hip motion [29]. In short, the increasing limitation of range of motion of the cervical spine impacts walking capacity. Although a limitation of this study is that the range of motion of the cervical spine was not assessed, it is possible in elderly patients with cervical fractures that limitation of the range of cervical spine motion due to fusion of more segments impacts walking capacity.

There are some possible limitations in the present study. Because of its retrospective design, there are some missing data. Because this was a retrospective multicenter study, the indications for surgery, choice of surgical technique, and postoperative treatment were left to the discretion of the surgeon at each hospital. Lack of detailed information such as surgeon, screw type, anesthesia and geriatric medical care, is also a limitation of this study. Although the follow-up rate (75%) was high, a selection bias could occur due to the retrospective investigation of only those patients who could be followed-up for 1 year. Because the sample size of the mortality group was small, a further study using a larger sample size is needed to better understand the factors that were associated with mortality after spinal fusion surgery for a cervical fracture in the elderly.

## Conclusions

The 1-year rate of mortality after spinal fusion surgery for cervical fracture in patients 65 years of age or older was 8%. A higher CCI score, a more severe AIS, and a longer surgical time were identified as independent factors associated with increasing 1-year mortality. The rate of deterioration in walking capacity between pre-trauma and 1 year postoperatively was 33%. A more severe AIS, lower Alb, lower Hb values, and a larger number of fused segments were identified as independent factors associated with an increasing risk of deterioration in walking capacity 1 year postoperatively.

## Data Availability

The datasets during and/or analyzed during the current study are available from the corresponding author on reasonable request.

## Abbreviations

JASA: Japan Association of Spine Surgeons with Ambition
CCI: Charlson comorbidity index
ASIA: American Spinal Cord Injury Association
AIS: American Spinal Cord Injury Association impairment scale
Alb: Albumin
Hb: Hemoglobin
OR: Odds ratio
CI: Confidence interval
